# Altered levels of cytokine, T- and B-lymphocytes, and PD-1 expression rates in drug-naïve schizophrenia patients with acute phase

**DOI:** 10.1038/s41598-023-49206-x

**Published:** 2023-12-07

**Authors:** Yali Zheng, Qi Zhang, Xianqin Zhou, Linjuan Yao, Quanfeng Zhu, Zhengchuang Fu

**Affiliations:** 1 Affiliated Xiaoshan Hospital, Hangzhou Normal University, Hangzhou, China; 2 Hangzhou Normal University, Hangzhou, China; 3 Hangzhou Di’an Medical Laboratory Center Co., Ltd, Hangzhou, China

**Keywords:** Immunology, Neuroscience, Diseases

## Abstract

Many studies have investigated the changes of immune cells and proinflammatory cytokines in patients with acute schizophrenia, but few studies have investigated the functional phenotypes of immune cells and the expression rate of programmed cell death protein 1 (PD-1)/ programmed cell death-Ligand 1 (PD-L1). The aim of this study was to investigate the extent of immune cells activation, PD-1/PD-L1 expressions, and altered cytokine levels in drug-naïve schizophrenia patients with acute-phase. 23 drug-naïve schizophrenia patients in acute-phase and 23 healthy individuals were enrolled in this study as experimental and control groups, separately. Socio-demographic information including gender, age, duration of illness, and smoking status was collected for each subject. Beckman DXFLEX triple laser thirteen-color flow cytometer and self-contained software CytoFLEX flow cytometric analysis software were used to detect the expressions of PD-1/PD-L1 on CD4+/CD8+ T lymphocytes, B lymphocytes, monocytes and NK cells. BD Bioscience was used to examine the levels of cytokines including interferon (IFN)-γ, tumor necrosis factor (TNF)-α, Interleukin (IL)-2, IL-4, IL-6, and IL-10. Drug-naïve schizophrenia patients in acute-phase had higher levels of peripheral blood CD4+ T lymphocytes and B lymphocytes, higher PD-1 expression in B lymphocytes, and lower levels of CD8+ T lymphocytes. In addition, IL-6 levels of peripheral blood were higher in schizophrenia patients (all *P* < 0.05). Significant immune stress was present in schizophrenia patients with acute-phase.

## Introduction

Schizophrenia is a severe form of psychosis with a high incidence, with a lifetime prevalence of up to 1%^1^. The most prevalent characteristic symptoms of schizophrenia are positive symptoms such as hallucinations, delusions, and disorganized speech, as well as negative symptoms such as decreased expressive ability, in addition, patients with schizophrenia also have varying degrees of cognitive impairment^2–4^. Current research has still not clarified the exact pathogenesis of schizophrenia, and environmental and genetic factors may play a crucial role in the development of schizophrenia. For example, results related to twin and family line studies suggest that genetic factors may explain 80% of the risk of schizophrenia^5^. Many people with schizophrenia have an onset in early adulthood, and the social and occupational impairments associated with illness follow them throughout their lifespan^6^. As a result, schizophrenia places a heavy burden on society. Schizophrenia can also lead to shorter life expectancy, with the average life expectancy of schizophrenia patients being statistically about 15 years shorter than healthy population^7^. Unfortunately, many patients do not achieve a satisfactory outcome after medication. Although study of the pathogenesis of schizophrenia has never stopped, but with the current treatment methods, there are still one-fifth to one-half of patients with no significant improvement in symptoms after treatment^8^.

The pathogenesis of schizophrenia is considered closely related to the dysfunction of glutamatergic neurotransmission, especially the dysfunction of endogenous N-methyl-d-aspartate (NMDA) receptor (NMDAR)^9,10^. Kynurenic acid, a metabolite of the kynurenic acid pathway, has antagonistic effects on NMDAR, and the increase of kynurenic acid levels in brain has been proved to reduce the release of dopamine and glutamate. Therefore, controlling the production of kynurenic acid in brain, especially kynurenine, is considered to be a promising target for the treatment of schizophrenia^11,12^. Studies have confirmed that the Kynurenic acid pathway plays a key role in the dysregulation of the immune system in schizophrenia, and the concentration of some key metabolites in the kynurenic acid pathway is altered in schizophrenia. Therefore, some views suggest that the metabolism disorder of kynurenic acid may be one of the reasons for the imbalance of cytokines in schizophrenia and lead to the occurrence and progression of the disease^13,14^. In addition, it has been confirmed that the activation of some cytokines may be associated with alterations in the kynurenic acid pathway^15^. Major histocompatibility complex (MHC) locus is also thought to play an important role in the regulation of immune system in schizophrenia, and its complex interaction with cytokines and other immune system regulators may be a potential driver of the immune hypothesis in schizophrenia^16^. The association of infection with schizophrenia and the increased incidence of autoimmune and inflammatory diseases in schizophrenia are strong evidence that MHC locus plays a role in schizophrenia^17^.

Several previous studies have reported the role of biological factors, particularly neuroimmunity, in the onset and progression of psychiatric disorders. For example, studies have shown that inflammatory factors can modulate signaling pathways in brain by affecting the secretion of neurotransmitters of brain, altering cognitive function, producing a range of mental and emotional symptoms, and even affecting the efficacy of medications^18^. Immune dysfunction is thought to play a pathogenic role in schizophrenia, and many studies have found that the concentration of peripheral inflammatory factors including cytokines is altered in patients with schizophrenia. Halstead et al. 's meta-analysis showed that elevated levels of Interleukin (IL)-6, IL-8, IL-10, and tumor necrosis factor (TNF)-α were found in both acute and chronic schizophrenia. In addition, the levels of IL-2 and interferon (IFN)-γ were significantly increased in patients with acute schizophrenia, while IL-4, IL-12 and Ifn-γ were significantly decreased in patients with chronic schizophrenia^19^. Our previous study investigated the changes of peripheral plasma cytokine levels including IL-2, IL-4, IL-6, IL-10, IL-17A, TNF-α, and IFN-γ in patients with bipolar disorder or major depression, and we found that both patients had significantly higher plasma IL-6 levels than healthy people^20^. A meta-analysis by Miller et al. found that IL-1β, IL-6, and transforming growth factor-β (TGF-β) may be markers of acute exacerbations of schizophrenia, whereas IL-12, IFN-γ, TNF-α may be trait markers^21^. In addition, several studies have shown the altered concentrations of pro-inflammatory cytokines including IL-2, IL-6, and IFN-γ in peripheral blood of patients with first-episode schizophrenia^22^. Some studies have also found alterations in the number of immune cells in peripheral blood of patients with schizophrenia^23,24^. However, due to various reasons such as comorbid disorders, use of antipsychotic medications, and some other reasons, the results obtained in many studies are not consistent, and therefore, the immune hypothesis of schizophrenia still needs more studies to be tested.

Existing studies have focused more on altered levels of immune cells, inflammatory factor expression and secretion in schizophrenia, but have rarely addressed the impact of functional phenotype of immune cells, such as lymphocytes, on psychiatric disorders. The purpose of our research was to investigate the alterations in functional phenotype of immune cells and the rate of PD-1/PD-L1 expression in immune cells in drug-naïve schizophrenia patients with acute-phase.

## Methods

### Recruitment of subjects

This project was approved by the Ethics Committee of Affiliated Xiaoshan Hospital, Hangzhou Normal University. Each subject was told the exact process of study and have signed a written informed consent form. In addition, all operations of this study were performed in accordance with the Declaration of Helsinki and in accordance with relevant guidelines/regulations. The inclusion criteria for experimental group in this study were: (1) meeting the Diagnostic and Statistical Manual of Mental Disorders-IV (DSM-IV) diagnostic criteria for schizophrenia; (2) first-episode and drug-naïve patients or patients with medication washout period of three months or more. The exclusion criteria for the experimental group were: (1) patients with diagnosis of diabetes, hypertension or other serious cardiovascular disease; (2) pregnant or lactating women; (3) patients with histories of acute infection in 3 months prior to study entry; (4) diagnosis of autoimmune disease or use of immunomodulatory drugs within 6 months before study initiation.

### Sociodemographic information collection

We collected socio-demographic information including age, marital status, education, disease duration, and smoking history of each subject by case finding and questioning.

### Measurement of cell surface markers

#### Collection of blood samples

All subjects were asked to fast after 10:00 p.m. the night before blood collection and to complete venous blood collection by 8:00 a.m. that morning. All blood samples were collected with EDTA-K2 vacuum blood collection tubes.

#### Blood specimen processing

100 μL of anticoagulated whole blood collected by EDTA-K2 vacuum blood collection tube was taken into a BD special test tube, with corresponding antibodies added, mixed evenly, kept away from light, and left at room temperature for 15 min. Then each tube was spiked with 1000 μL of BD matching lysate and left at room temperature for 10–15 min and then centrifuged at 500 ×*g* for 5 min. The supernatant was then discarded, 1000 μL of phosphate buffered saline (PBS) was added and centrifuged at 500 ×*g* for 5 min, and the washing operation was repeated once more. After discarding the supernatant again, the cells were resuspended with 500 μL PBS, mixed and assayed on the machine.

#### Sample analysis

Specimens in this study were examined using Beckman DXFLEX triple laser thirteen-color flow cytometer and experimental results were analyzed by self-contained software CytoFLEX flow cytometric analysis software. Beckman calibration quality control microspheres were used to calibrate instrument optical pathway system, and isotype controls were used to set positivity thresholds, with simultaneous gating using SSC/FSC and SSC/CD45. CD8, CD4, PD-1, CD14, PD-L1, CD19, CD3, CD56, and CD45 differentiation antigens were detected, and lymphocytes were circled by SSC/CD45 graphic, and then CD3+ T lymphocytes, CD3+CD4+ T lymphocytes, CD3+CD8+ T lymphocytes, CD3-CD56+NK cells, CD19+B lymphocytes, and CD14+ monocytes were circled and observed in each population of cells for PD-1 and PD-L1 expressions.

### Measurement of plasma cytokine

Levels of serum cytokines, including interferon IL-2, IL-4, IL-6, IL-10, IL-17A, TNF-α, and IFN-γ were measured by using BD cytometric bead array human Th1/Th2 cytokine kit (BD Bioscience). Through the use of standard curves created with each of the given recombinant cytokines, the concentrations of serum cytokines were ascertained. The results of cytokine assays were based on the fitting of a standard curve, with 2.3 pg/mL and 5000 pg/mL were the lowest and highest values of the standard cu the results of cytokine assays were based on the fitting of a standard curve, with 2.3 pg/mL and 5000 pg/mL were the lowest and highest values of the standard curve, respectively. To ensure the accuracy of the results, the above assay was repeated three times. The laboratory personnel responsible for data interpretation and report issuance were professional technicians with formal technical training and some clinical experience.

### Statistical analysis

The data were categorized into schizophrenia and control groups, and normality of all continuous variables was tested using Shapiro–Wilk test. Then T-Test was used for continuous variables that conformed to normal distribution, Mann–Whitney U Test was used for continuous and ranked variables that did not conform to normal distribution, and Chi-Square Test was used for categorical variables. In order to exclude the influence of gender, age, and smoking on levels of immune cells and cytokines, we used gender, age, and smoking status as covariates, and variables with significant differences after above univariate analysis were used as independent variables for binary logistic regression analysis. In addition, we also analyzed the effects of gender, smoking, and marital status on cytokine levels separately in experimental group using the univariate analysis method described above. All statistical analyses were done on SPSS 25.0 software. In the present study, two-tailed *P* values < 0.05 were considered significant.

## Results

### Demographic characteristics

The subjects were initially screened according to the inclusion and exclusion criteria, and then 4 subjects were excluded in total with white blood cells, neutrophils, or C-reactive protein levels higher than baseline levels. Finally, a total of 23 patients with acute schizophrenia, of whom 4 were first-episode and drug-naïve and the other 19 patients had a drug washout period of more than 3 months, and 23 healthy controls were recruited in this study. As shown in Table 1, in schizophrenia group, there were 13 males and 10 females, 11 unmarried and 12 married, 12 smokers and 11 non-smokers, the age distribution was 40.17 ± 12.63 years, and the disease duration distribution was 11.29 ± 8.32 years. In healthy control group, there were 6 males and 17 females, 15 unmarried and 8 married, 6 smokers and 17 non-smokers with an age distribution of 38.65 ± 10.51 years. No discernible difference existed between two groups on above socio-demographic information (all *P* > 0.05).

**Table 1:** Socio-demographic information of experimental and healthy control groups.

Variable	Experimental group	Healthy control group	t/χ^2^/Z	*P*
Age	40.17 ± 12.63	38.65 ± 10.51	0.44	0.66
Gender			4.39	0.07
Male, n (%)	13 (56.5%)	6 (26.1%)		
Female, n (%)	10 (43.5%)	17 (73.9%)		
Marital status			1.42	0.37
Unmarried, n (%)	11 (47.8%)	15 (65.2%)		
Married, n (%)	12 (52.2%)	8 (34.8%)		
Smoking			3.29	0.13
Yes, n (%)	12 (52.2%)	6 (26.1%)		
No, n (%)	11 (47.8%)	17 (73.9%)		

C ontinuous variables conforming to normal distribution: mean ± standard deviation.

Continuous variables that do not conform to normal distribution: median (25 percentile quantile, 75 percentile quantile).

### Activation pattern of T-, B-lymphocytes, NK cells, and monocytes

The peripheral blood was analyzed for the percentage of T-, B-lymphocytes, monocytes, and NK cells, as well as the percentage of CD4+ and CD8+ T- lymphocytes. According to Table 2, the percentage of CD4+ T lymphocytes and B lymphocytes in peripheral blood of schizophrenia patients was significantly higher, whereas the percentage of CD8+ T lymphocytes was significantly lower than in healthy population (all *P* < 0.05). Also, patients with schizophrenia had significantly higher CD4+/CD8+ ratio (*P* < 0.01).

**Table 2:** Distribution and activation in immune cells of experimental and healthy control groups.

Variable	Experimental group	Healthy control group	t/χ^2^/Z	*P*
T lymphocytes (%)	66.81 ± 9.54	70.14 ± 7.18	− 1.34	0.19
CD4+ T lymphocytes (%)	56.73 ± 10.65	48.92 ± 10.33	2.53	0.02*
CD8+ T lymphocytes (%)	35.08 ± 10.01	40.38 ± 7.04	− 2.08	0.04*
CD4+/CD8+ ratio	1.81 ± 0.79	1.28 ± 0.43	2.88	0.01**
B lymphocytes (%)	15.43 ± 5.48	11.73 ± 3.44	2.74	0.01**
Monocytes (%)	6.01 ± 1.51	6.15 ± 2.14	− 0.26	0.80
NK cells (%)	14.01(9.24–22.94)	14.47(9.22–21.48)	− 0.19	0.86

*Significant at *P* < 0.05.

**Significant at *P* < 0.01.

Continuous variables conforming to normal distribution: mean ± standard deviation.

Continuous variables that do not conform to normal distribution: median (25 percentile quantile, 75 percentile quantile).

### Expression of immune checkpoint proteins

We explored PD-1/PD-L1 expression levels of various immune cells in patients with schizophrenia as well as in healthy controls. As shown in Table 3, PD-1 expression rate of B lymphocytes was significantly higher in patients with schizophrenia than in healthy population (*P* < 0.01).

**Table 3:** PD-1/PD-L1 expression in immune cells of experimental and healthy control groups.

Variable	Experimental group	Healthy control group	t/χ^2^/Z	*P*
CD4+ T lymphocytes PD-1 expression (%)	14.68(9.25–22.89)	19.35(10.34–25.95)	− 0.19	0.46
CD4+ T lymphocytes PD-L1 expression (%)	41.15(5.96–68.14)	25.93(9.29–56.92)	− 0.54	0.60
CD8+ T lymphocytes PD-1 expression (%)	21.23 ± 8.87	17.29 ± 8.98	1.50	0.14
CD8+ T lymphocytes PD-L1 expression (%)	11.78(2.9–34.29)	13.13(4.62–30.82)	− 0.04	0.97
B lymphocytes PD-1 expression (%)	3.59 ± 2.27	1.85 ± 0.87	3.42	0.002**
B lymphocytes PD-L1 expression (%)	7.75(2.97–24.44)	7.43(4.36–12.29)	− 0.32	0.76
Mononuclear cells PD-1 expression (%)	0.06(0.04–0.14)	0.04(0.03–0.06)	− 1.88	0.06
Mononuclear cells PD-L1 expression (%)	0.04(0.01–0.29)	0.07(0.01–0.45)	− 0.17	0.87
NK cells PD-1 expression (%)	0.36(0.06–0.99)	0.13(0.09–0.21)	− 1.44	0.15
NK cells PD-L1 expression (%)	3.06(0.8–6.81)	3.65(1.04–12.59)	− 0.69	0.50

**Significant at *P* < 0.01.

Continuous variables conforming to normal distribution: mean ± standard deviation.

Continuous variables that do not conform to normal distribution: median (25 percentile quantile, 75 percentile quantile).

### Plasma level of cytokines

Cytokine levels including IL-2, IL-4, IL-6, IL-10, TNF-α, and IFN-γ were tested, and we found significantly higher levels of IL-6 in patients with schizophrenia (*P* < 0.01) (Table 4). In addition, as shown in Table S1–S5, in patients with acute schizophrenia, the levels of cytokines were not significantly correlated with gender, smoking, and marital status, while the level of IL-4 was significantly negatively correlated with age (r = − 0.47, *P* = 0.02) and disease duration (r = − 0.51, *P* = 0.01).

**Table 4:** Cytokine levels of experimental and healthy control groups.

Variable	Experimental group	Healthy control group	t/χ^2^/Z	*P*
IL-2, pg/mL	1.51 ± 0.53	1.56 ± 0.41	− 0.38	0.71
IL-4, pg/mL	0.99(0.82–1.06)	0.99(0.85–1.1)	− 0.14	0.89
IL-6, pg/mL	1.91(1.57–3.17)	1.43(1.32–1.87)	− 2.75	0.01**
IL-10, pg/mL	1.37(1.22–1.64)	1.49(1.29–1.67)	− 0.53	0.61
IL-17A, pg/mL	0.48(0.17–0.79)	0.44(0.26–0.73)	− 0.31	0.77
TNF-α, pg/mL	1.22(0.89–1.51)	1.33(0.92–1.65)	− 0.43	0.68
IFN-γ, pg/mL	0.69(0.57–0.77)	0.74(0.61–0.86)	− 1.01	0.32

Continuous variables conforming to normal distribution: mean ± standard deviation.

Continuous variables that do not conform to normal distribution: median (25 percentile quantile, 75 percentile quantile).

** Significant at *P* < 0.01.

### The influence of confounding factors

After binary logistic regression analysis with gender, age and smoking as covariates, the results showed that the percentage of CD4+ T lymphocytes (*P* = 0.002, B = 0.15, adjusted OR 1.16, 95% CI 1.06–1.28), B lymphocytes (*P* = 0.014, B = 0.22, adjusted OR 1.25, 95% CI 1.05–1.49) and the PD-1 expression rate of B lymphocytes (*P* = 0.008, B = 0.80, adjusted OR 2.23, 95% CI 1.23–4.05) in patients with acute schizophrenia were still significantly higher than those in healthy population, and the percentage of CD8+ T lymphocytes (*P* = 0.008, B = − 0.13, adjusted OR 0.88, 95% CI 0.79–0.97) was still significantly lower. However, the difference in IL-6 levels (*P* = 0.053, B = 1.19, adjusted OR 3.29, 95% CI 0.99–11.01) between patients with acute schizophrenia and healthy population was no longer significant.

## Discussion

This study explored the distribution and expression of immune cells and secretion of cytokines in peripheral blood of drug-naïve schizophrenia patients with acute-phase. The main findings of this study were as follows: (1) patients with schizophrenia had higher levels of peripheral blood B lymphocytes and CD4+ T lymphocytes, higher PD-1 expression of B lymphocytes, and lower levels of CD8+ T lymphocytes; (2) the level of IL-6 in peripheral blood was higher in patients with acute schizophrenia, but the results were no longer significant after adjusting for confounding factors such as gender, age and smoking.

To date there have been many similar studies that sought to explore the relationship between immune function and schizophrenia through peripheral immune markers^25–27^, and much evidence confirms that dysregulation of immune system has a significant impact on onset and progression of schizophrenia or other psychiatric disorders^28,29^. However, many schizophrenia patients in previous studies had comorbidities with different diseases and were on different antipsychotic medications, and these factors would interfere with results of those studies. In our study, all subjects in experimental group were drug-naïve schizophrenia patients in acute-phase or acute patients with medication washout period of three months or more; in addition, we excluded patients with hypertension, diabetes mellitus, or other serious cardiovascular diseases and those who were on immunosuppressive, immunopotentiator therapies, or who had immunodeficiencies.

As we all know, B lymphocytes dominate humoral immunity in our body, while T lymphocytes dominate cellular immunity, B and T lymphocytes coordinate and interact with each other to maintain immune environment^30,31^. In the current research, we discovered that schizophrenia patients with acute-phase had significantly higher B-lymphocyte ratios in peripheral blood than healthy population. In addition, patients had significantly higher CD4+ T lymphocytes ratios and lower CD8+ T lymphocytes ratios than healthy population, which were similar to the findings of some other studies^32^. It has been suggested that CD4/CD8 ratio may be a state marker of acute exacerbation of schizophrenia, because the results of a meta-analysis showed that CD4/CD8 ratio was significantly increased in first-episode schizophrenia patients, while it was significantly decreased after drug treatment^33^. However, there are various shortcomings in the studies involved, for example, some studies did not consider the interference caused by gender, age, smoking and other factors. In this study, after excluding the confounding factors of gender, age, smoking and antipsychotic drugs, the CD4/CD8 ratio of patients with acute schizophrenia was significantly increased. This result is consistent with the macrophage-T-lymphocyte theory of the schizophrenia immune hypothesis, which suggests that cytokines produced by chronically activated macrophages and T lymphocytes are fundamental mediators of schizophrenia^34^.

IL-6 is a kind of pro-inflammatory cytokines, is significantly increased in acute inflammatory states, and is important for stimulation and differentiation of B lymphocytes^35^. Although alterations in peripheral cytokines such as IL-6 do not necessarily fully represent the state of central immune response, elevated levels of IL-6 have been found to be present in acute phase of multiple psychiatric disorders, including schizophrenia, bipolar disorder, and major depressive disorder, and have even been suggested to be a possible state marker for schizophrenia^36^, this suggests a common immune stress-related phenomenon in acute phase of schizophrenia or even other psychiatric disorders^37^. Several studies have also found that IL-6-related pathways are critical for brain development, and that abnormally elevated levels of IL-6 may affect the development of brain regions closely associated with neuropsychiatric disorders^38^. It should be noted that the levels of immune cells and cytokines in peripheral blood are affected by multiple factors, such as obesity and impaired glucose tolerance^39^, and few studies have completely eliminated all these confounding factors. In this study, IL-6 levels were elevated but not above baseline levels in patients with acute schizophrenia, and the difference was not statistically significant after excluding confounding factors. Therefore, we cannot conclude that peripheral blood cytokine levels are altered in patients with acute schizophrenia, and caution is needed as biomarkers for schizophrenia.

This study also identified a significantly higher rate of PD-1 expression in B lymphocytes of schizophrenia patients with acute-phase. The interaction of PD-1 and its ligands plays a key role in some diseases, such as autoimmune diseases, infectious diseases, and tumors^40–43^. In schizophrenia, however, PD-1 expression has been less frequently mentioned or studied. PD-1 is an inhibitory co-stimulatory molecule for immune cells and has important negative regulatory effects on immune cell function^44,45^. We suggest that high expression of PD-1 in B lymphocytes of patients in this study may be related to high expression of other immune cells and that it may play a role in avoiding the depletion of other regulatory immune cells. Due to the lack of relevant evidence, the role played by PD-1 expression in schizophrenia or other psychiatric disorders needs to be confirmed by more studies.

Our research has several limitations. First, the sample size of this study was not based on a priori power analyses, which was small due to funding constraints, it would weaken the reliability of our statistical results. Second, we did not repeat the study after patients’ symptoms had resolved, which prevented us from exploring the differences in immune cell distribution, expression, and cytokine levels between patients in acute and remission phases.

In conclusion, our study confirmed that there are certain characteristic changes in immune cells in patients with acute schizophrenia, such as an increase in CD4/CD8 ratio, which can provide some support for the immune hypothesis of schizophrenia.

### Supplementary Information

Supplementary Information 1.

Supplementary Information 2.

Supplementary Information 3.

Supplementary Information 4.

Supplementary Information 5.

Supplementary Information 6.

## Data Availability

The raw data that support the findings of this study has been provided in Supplementary Material, but permission to use our data for research and publication should be obtained from the corresponding author first.
