# Supplementary material for: Altered levels of cytokine, T- and B-lymphocytes, and PD-1 expression rates in drug-naïve schizophrenia patients with acute phase
Source: Sci Rep. 2023 Dec 7;13:21711. doi: 10.1038/s41598-023-49206-x (PMC10709554; doi:10.1038/s41598-023-49206-x)
Supplement: Supplementary file 2 — Supplementary Information 2. [file 41598_2023_49206_MOESM2_ESM.docx]

Table S1: Effect of gender on cytokine levels in patients with acute schizophrenia

| **Variable** | **Male** | **Female** | **t/χ2/Z** | **P** |
| --- | --- | --- | --- | --- |
| IL-2, pg/mL | 1.41±0.46 | 1.64±0.61 | -1.05 | 0.31 |
| IL-4, pg/mL | 0.97±0.19 | 0.93±0.25 | 0.37 | 0.71 |
| IL-6, pg/mL | 2.13(1.67~3.34) | 1.88(1.33~3.01) | -0.93 | 0.35 |
| IL-10, pg/mL | 1.24±0.47 | 1.56±0.28 | -1.96 | 0.06 |
| IL-17A, pg/mL | 0.50±0.33 | 0.54±0.43 | -0.23 | 0.82 |
| TNF-α, pg/mL | 1.12(0.91~1.65) | 1.36(0.89~1.48) | 0 | 1 |
| IFN-γ, pg/mL | 0.68(0.56~0.86) | 0.71(0.60~0.74) | -0.03 | 0.98 |

Continuous variables conforming to normal distribution: mean ± standard deviation

Continuous variables that do not conform to normal distribution: median (25 percentile quantile, 75 percentile quantile)
