# Supplementary material for: Altered levels of cytokine, T- and B-lymphocytes, and PD-1 expression rates in drug-naïve schizophrenia patients with acute phase
Source: Sci Rep. 2023 Dec 7;13:21711. doi: 10.1038/s41598-023-49206-x (PMC10709554; doi:10.1038/s41598-023-49206-x)
Supplement: Supplementary file 3 — Supplementary Information 3. [file 41598_2023_49206_MOESM3_ESM.docx]

Table S2: Effect of smoking on cytokine levels in patients with acute schizophrenia

| **Variable** | **Smoker** | **Nonsmoker** | **t/χ2/Z** | **P** |
| --- | --- | --- | --- | --- |
| IL-2, pg/mL | 1.48±0.55 | 1.54±0.53 | -0.24 | 0.81 |
| IL-4, pg/mL | 1.02±0.23 | 0.87±0.18 | 1.71 | 0.10 |
| IL-6, pg/mL | 2.27(1.73~3.43) | 1.85(1.35~2.25) | -1.66 | 0.10 |
| IL-10, pg/mL | 1.30±0.52 | 1.47±0.27 | -0.97 | 0.34 |
| IL-17A, pg/mL | 0.50(0.28~0.76) | 0.25(0.10~0.97) | -0.22 | 0.83 |
| TNF-α, pg/mL | 1.43(1.01~1.67) | 1.05(0.89~1.43) | -1.36 | 0.18 |
| IFN-γ, pg/mL | 0.66(0.55~0.78) | 0.70(0.62~0.77) | -0.37 | 0.71 |

Continuous variables conforming to normal distribution: mean ± standard deviation

Continuous variables that do not conform to normal distribution: median (25 percentile quantile, 75 percentile quantile)
