# Supplementary material for: Altered levels of cytokine, T- and B-lymphocytes, and PD-1 expression rates in drug-naïve schizophrenia patients with acute phase
Source: Sci Rep. 2023 Dec 7;13:21711. doi: 10.1038/s41598-023-49206-x (PMC10709554; doi:10.1038/s41598-023-49206-x)
Supplement: Supplementary file 4 — Supplementary Information 4. [file 41598_2023_49206_MOESM4_ESM.docx]

Table S3: Effect of marital status on cytokine levels in patients with acute schizophrenia

| **Variable** | **Married** | **Unmarried** | **t/χ2/Z** | **P** |
| --- | --- | --- | --- | --- |
| IL-2, pg/mL | 1.68±0.53 | 1.33±0.49 | 1.65 | 0.11 |
| IL-4, pg/mL | 0.91±0.23 | 0.99±0.20 | -0.88 | 0.39 |
| IL-6, pg/mL | 1.88(1.42~2.60) | 2.13(1.68~3.51) | -0.92 | 0.36 |
| IL-10, pg/mL | 1.54±0.28 | 1.21±0.49 | 2.00 | 0.06 |
| IL-17A, pg/mL | 0.57±0.41 | 0.46±0.32 | 0.75 | 0.47 |
| TNF-α, pg/mL | 1.36(0.93~1.46) | 1.12(0.67~1.69) | -0.22 | 0.83 |
| IFN-γ, pg/mL | 0.66±0.10 | 0.78±0.28 | -1.48 | 0.17 |

Continuous variables conforming to normal distribution: mean ± standard deviation

Continuous variables that do not conform to normal distribution: median (25 percentile quantile, 75 percentile quantile)
