# Supplementary material for: Altered levels of cytokine, T- and B-lymphocytes, and PD-1 expression rates in drug-naïve schizophrenia patients with acute phase
Source: Sci Rep. 2023 Dec 7;13:21711. doi: 10.1038/s41598-023-49206-x (PMC10709554; doi:10.1038/s41598-023-49206-x)
Supplement: Supplementary file 5 — Supplementary Information 5. [file 41598_2023_49206_MOESM5_ESM.docx]

Table S4: Association of age and cytokine levels in patients with acute schizophrenia

| **Variable** | **Correlation coefficient** | **P** |
| --- | --- | --- |
| IL-2, pg/mL | -0.06 | 0.80 |
| IL-4, pg/mL | -0.47 | 0.02^*^ |
| IL-6, pg/mL | 0.16 | 0.48 |
| IL-10, pg/mL | 0.05 | 0.82 |
| IL-17A, pg/mL | -0.002 | 0.99 |
| TNF-α, pg/mL | 0.07 | 0.76 |
| IFN-γ, pg/mL | -0.06 | 0.79 |

^*^ Significant at p *<* 0.05
^**^ Significant at p *<* 0.01
