# Supplementary material for: Altered levels of cytokine, T- and B-lymphocytes, and PD-1 expression rates in drug-naïve schizophrenia patients with acute phase
Source: Sci Rep. 2023 Dec 7;13:21711. doi: 10.1038/s41598-023-49206-x (PMC10709554; doi:10.1038/s41598-023-49206-x)
Supplement: Supplementary file 6 — Supplementary Information 6. [file 41598_2023_49206_MOESM6_ESM.docx]

Table S5: Association of disease duration and cytokine levels in patients with acute schizophrenia

| **Variable** | **Correlation coefficient** | **P** |
| --- | --- | --- |
| IL-2, pg/mL | -0.19 | 0.38 |
| IL-4, pg/mL | -0.51 | 0.01^*^ |
| IL-6, pg/mL | 0.05 | 0.83 |
| IL-10, pg/mL | 0.11 | 0.63 |
| IL-17A, pg/mL | -0.11 | 0.63 |
| TNF-α, pg/mL | -0.29 | 0.18 |
| IFN-γ, pg/mL | 0.04 | 0.86 |

^*^ Significant at p *<* 0.05
^**^ Significant at p *<* 0.01
